# Inhibition of CLIC4 Enhances Autophagy and Triggers Mitochondrial and ER Stress-Induced Apoptosis in Human Glioma U251 Cells under Starvation

**DOI:** 10.1371/journal.pone.0039378

**Published:** 2012-06-25

**Authors:** Jiateng Zhong, Xiaoxia Kong, Hongyu Zhang, Chunyan Yu, Ye Xu, Jinsong Kang, Huimei Yu, Haowei Yi, Xiaochun Yang, Liankun Sun

**Affiliations:** 1 Department of Pathophysiology, Norman Bethune College of Medicine, Jilin University, Changchun, Jilin, China; 2 Institute of Hypoxia Medical Research, Teaching Center of Functional Experiment, Basic Medical School, Wenzhou Medical College, Wenzhou, China; 3 School of Pharmacy, Wenzhou Medical College, Wenzhou, China; 4 Department of Pathology, Basic Medical College, Beihua University, Jilin, China; 5 Medical Research Laboratory, Jilin Medical College, Jilin, China; 6 Department of Pathogenobiology, Norman Bethune College of Medicine, Jilin University, Changchun, Jilin, China; University of Campinas, Brazil

## Abstract

CLIC4/mtCLIC, a chloride intracellular channel protein, localizes to mitochondria, endoplasmic reticulum (ER), nucleus and cytoplasm, and participates in the apoptotic response to stress. Apoptosis and autophagy, the main types of the programmed cell death, seem interconnected under certain stress conditions. However, the role of CLIC4 in autophagy regulation has yet to be determined. In this study, we demonstrate upregulation and nuclear translocation of the CLIC4 protein following starvation in U251 cells. CLIC4 siRNA transfection enhanced autophagy with increased LC3-II protein and puncta accumulation in U251 cells under starvation conditions. In that condition, the interaction of the 14-3-3 epsilon isoform with CLIC4 was abolished and resulted in Beclin 1 overactivation, which further activated autophagy. Moreover, inhibiting the expression of CLIC4 triggered both mitochondrial apoptosis involved in Bax/Bcl-2 and cytochrome c release under starvation and endoplasmic reticulum stress-induced apoptosis with CHOP and caspase-4 upregulation. These results demonstrate that CLIC4 nuclear translocation is an integral part of the cellular response to starvation. Inhibiting the expression of CLIC4 enhances autophagy and contributes to mitochondrial and ER stress-induced apoptosis under starvation.

## Introduction

Apoptosis and autophagy (also referred to as programmed cell death types I and II, respectively) are the most common forms of programmed cell death [1], [2]. Apoptosis is thought to involve the activation of caspases and a stereotyped sequence of mitochondrial alterations [3]. In contrast to apoptosis, autophagy is characterized by the presence of autophagic vesicles (autophagosomes) that engulf bulk cytoplasm and cytosolic organelles such as the endoplasmic reticulum (ER) and mitochondria, with subsequent degradation by the cell lysosomal system [4], [5]. The outcome of activating the autophagy program is highly dependent on the cellular context, strength and duration of stress-inducing signals [6], [7], [8]. Thus, besides its role in cellular homeostasis, autophagy can play a cytoprotective role, for example in situations of nutrient starvation [9]. Accordingly, autophagy plays an important role in both tumor progression and promotion of cancer cell death [10], [11], although the molecular mechanism responsible for this dual action of autophagy is unclear. In addition, the relationship between autophagy and apoptosis is complex and varies with cell type, specific extrinsic stresses, the addition of certain activators or inhibitors [10], [12], [13] or regulation of relative proteins by molecular strategies [12]. In addition, recent findings have shown a role for ER stress in regulating autophagy and cell death, but the underlying mechanism remains to be characterized.

CLIC4 belongs to the chloride intracellular channel (CLIC) family of proteins, the most studied of the seven highly homologous members [14], [15]. Reports on the subcellular localization of CLIC4 *in vitro* still do not form a coherent pattern; CLIC4 seems localized in the cytoplasm, mitochondria [16], ER membrane, in large dense core vesicles in neurons, and in the nucleus [14]. It is likely that changes in the subcellular localization of CLIC4 are critical in the regulating its function. An intriguing aspect of CLIC4 biology is its role as an effector of apoptosis, including p53 and c-Myc-induced apoptosis, as well as in response to cytotoxic and genotoxic stresses. Cytoplasmic CLIC4 translocates to the nucleus under conditions of stress mediated by a functional nuclear localization signal (NLS) on the carboxy terminus of the protein [17]. Nuclear CLIC4 residence is an essential component of its pro-apoptotic and growth arrest activity in keratinocytes [16]. Furthermore, CLIC4 contains several binding domains that interact with proteins near the NLS, including α-tubulin, dynamin 1 and the 14-3-3 protein family [18]. The physiologic function of CLIC4 has been implicated in regulating cell cycle arrest, apoptosis, metabolic stress, cell differentiation, morphogenesis, and a novel molecular target for cancer therapy [14].

Despite being multifunctional, the role of CLIC4 in autophagy has yet to be investigated. In the present study, we demonstrated that, U251 cells under starvation conditions caused upregulation and nuclear translocation of the CLIC4 protein, while inhibition of CLIC4 by siRNA enhanced autophagy. The results indicate the role of CLIC4 in autophagy is related to its interaction with the 14-3-3 epsilon protein and increased expression of the autophagic protein Beclin 1. Inhibition of CLIC4 by siRNA under starvation conditions triggered both mitochondrial apoptosis involved in the Bcl-2/Bax and caspase-3 pathway and ER stress-induced apoptosis with CHOP and caspase-4 upregulation.

## Results

### Starvation Induces Autophagy but not Apoptosis in Human Glioma U251 Cells

**Figure 1:**
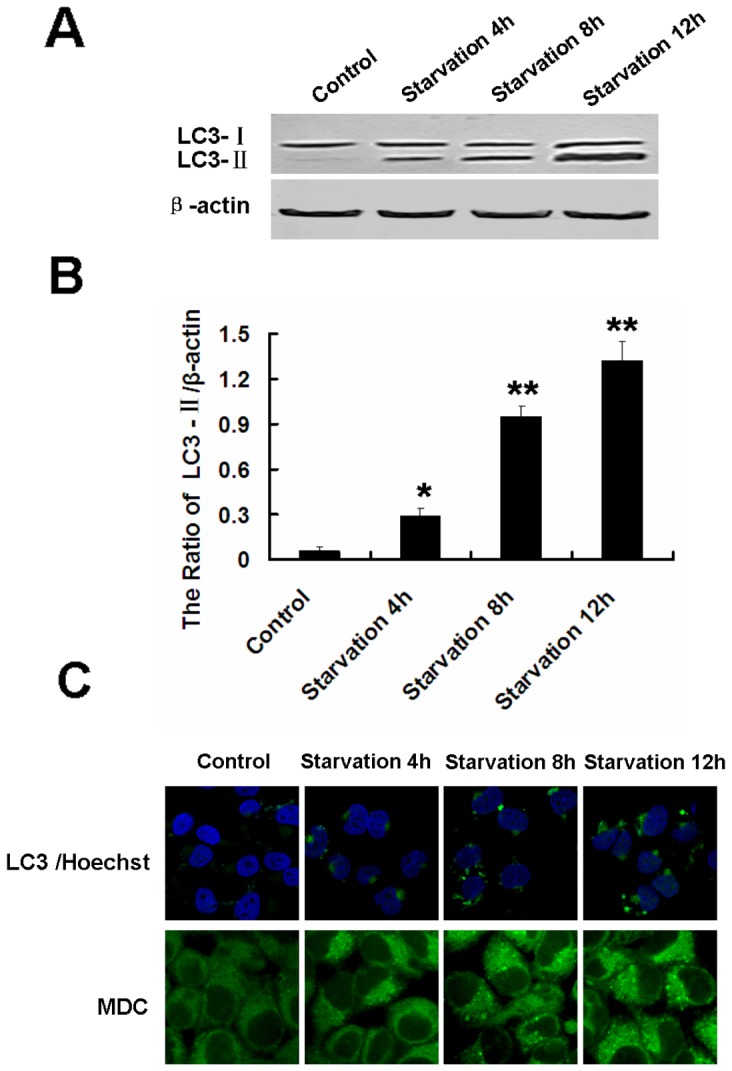
Starvation-induced autophagy in human glioma U251 cells. (A) Western blot analysis of LC3 expression in U251 cells incubated with EBSS for 4 h, 8 h and 12 h. (B) The ratio of LC3-II/β-actin. Data were presented as a mean ± SD of three independent experiments. ^*^ *P*<0.05 and ^*^ *P*<0.01 versus control group. (C) Immunostaining of LC3 puncta (Hoechst 33342 staining for the nucleus, 600×) and MDC (900×) were observed by confocal microscopy in U251 cells after incubation with EBSS for 4 h, 8 h and 12 h.

**Figure 2:**
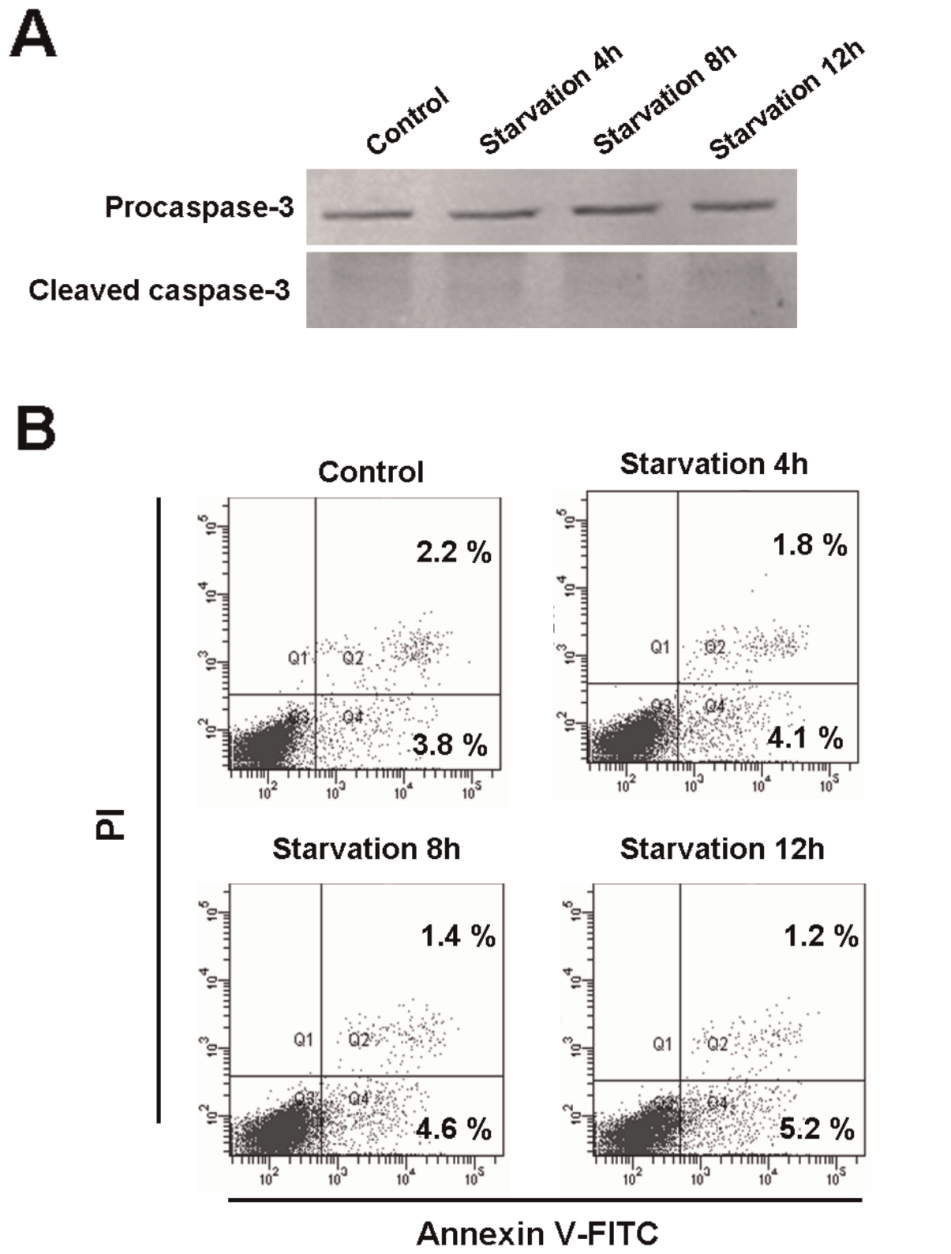
Starvation does not induce apoptosis in human U251 cells. (A) Western blot analysis of Procaspase-3 and Cleaved caspase-3 expression in U251 cells incubated with EBSS for 4 h, 8 h and 12 h. (B) Flow cytometric analysis of apoptosis in U251 cells incubated with EBSS for 4 h, 8 h and 12 h. Cells were stained with PI and Annexin V-FITC. The positive stained cells were counted using FACScan. Data were presented as mean, n = 3.

For studies of autophagy under amino acid starvation, glioma U251 cells were incubated in EBSS at different time points. During starvation, microtubule associated protein LC3 (the mammalian equivalent of yeast Atg8) localized to isolation membranes leading to the formation of autophagosome membranes. Therefore, detection of a punctuated pattern of cytosolic LC3 indicates involvement of LC3 in autophagosome formation; a phenomenon used to monitor autophagy. LC3 exists in two cellular forms, LC3-I (18 kDa) and LC3-II (16 kDa). LC3-I converts to LC3-II by conjugation of phosphatidylethanolamine and the amount of LC3-II becomes a marker for the formation of autophagosomes [19], [20]. In addition, LC3 exhibits a puncta distribution following induced autophagy [21]. We demonstrate the expression of LC3-II was significantly increased in a time-dependent manner (Fig. 1A and 1B). Cells incubated with EBSS for 4 h, 8 h and 12 h developed increased LC3 puncta distribution, indicating increased autophagosomes in the cytoplasm when compared with the control group (Fig. 1C upper line; nuclei stained with Hoechst 33342). The cell autophagic level was determined by MDC staining, as MDC accumulates in mature autophagic structures, the bright dots in the staining indicate autophagosomes [22]. MDC staining showed autophagy activation following EBSS incubation, results consistent with the LC3 analysis (Fig. 1C). To further investigate whether apoptosis was also involved in cell death under starvation conditions, caspase-3 expression was analyzed. No evidence of cleaved caspase-3 expression was present at any time point (Fig. 2A). PI and Annexin V-FITC staining also determined the cell apoptotic rate. FCM results indicated there was no significant variation in apoptotic rate among the 4 h, 8 h and 12 h starvation groups, compared with the control group (Fig. 2B).

### Starvation-induced CLIC4 Upregulation and Nuclear Translocation in Glioma U251 Cells

**Figure 3:**
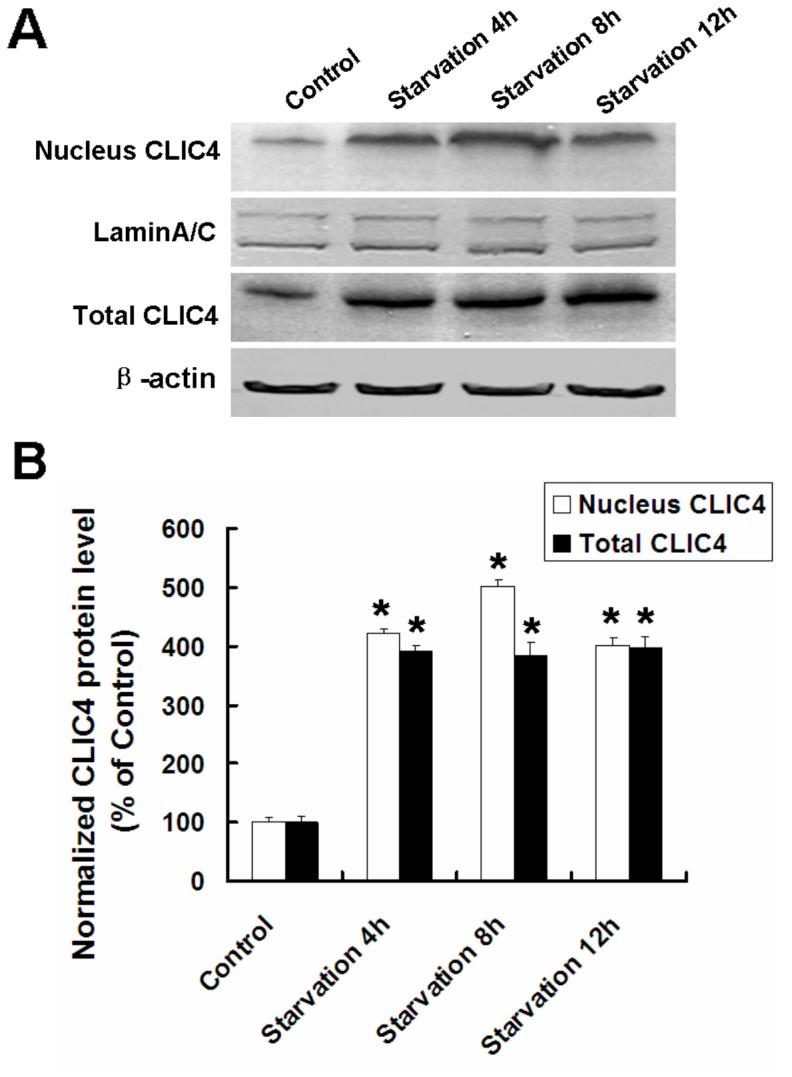
CLIC4 upregulated and nuclear translocated in U251 cells under starvation conditions. (A) Western blot analysis of nuclear CLIC4 and total CLIC4 expressions in U251 cells incubated with EBSS for 4 h, 8 h and 12 h. (B) Densitometric analysis of normalized nuclear CLIC4 and total CLIC4 protein levels. Standard error represents three independent experiments. Data were presented as mean ± SD. ^*^ *P*<0.05 versus control group.

As a protein predominantly expressed in the mitochondria and ER, CLIC4 is essential for p53 and c-Myc-mediated apoptosis. CLIC4 nuclear translocation under stress has been reported, however whether CLIC4 is associated with starvation stress is unknown. Total CLIC4 and nuclear CLIC4 increased in U251 cells cultured with EBSS, compared with the control group (Fig. 3A and 3B), suggesting that CLIC4 may be a potential responder or regulator under starvation conditions.

### Partial Inhibition of CLIC4 by siRNA Enhances Autophagy and Triggers Apoptosis Under Starvation Conditions

**Figure 4:**
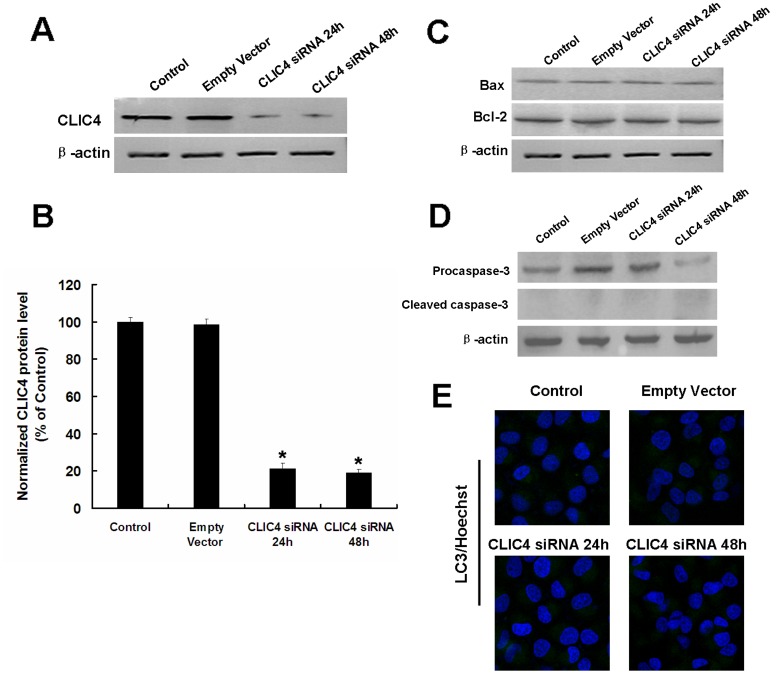
The effect of CLIC4 siRNA on apoptosis and autophagy in U251 cells. (A) Western blot analysis of CLIC4 expression in U251 cells transfected with a plasmid encoding empty vector or CLIC4 siRNA vector for 24 h and 48 h. (B) Densitometric analysis of CLIC4 levels. Data were presented as a mean ± SD of three independent experiments. ^*^ *P*<0.05 versus control group. (C) Western blot analysis of Bax and Bcl-2 expressions in U251 cells transfected with a plasmid encoding empty vector or CLIC4 siRNA vector for 24 h and 48 h. (D) Western blot analysis of Procaspase-3 and Cleaved caspase-3 expressions in U251 cells transfected with a plasmid encoding empty vector or CLIC4 siRNA vector for 24 h and 48 h. (E) LC3 immunostaining (Hoechst 33342 staining for the nucleus, 600×) were observed by confocal microscopy in U251 cells transfected with a plasmid encoding empty vector or CLIC4 siRNA vector for 24 h and 48 h.

To further confirm the role of CLIC4 under starvation conditions, two CLIC4 siRNA plasmids (CLIC4-siRNA and CLIC4-siRNA-S) were constructed and transiently transfected into U251 cells. According to the efficiency of transfection, we chose CLIC4-siRNA (Figure S1A and S1B). CLIC4 siRNA transfection inhibited the expression of CLIC4 protein significantly in U251 cells after 24 h (Fig. 4A and 4B). Because the extensive sequence homology among the CLICs, we detected the expression of other two CLICs CLIC1 and CLIC5 which are closely related to CLIC4. There was no significant change of these two CLICs under CLIC4 siRNA transfection (Figure S2). The effects of CLIC4 siRNA transfection on cell apoptosis and autophagy were assessed. There was no significant difference in the expressions of apoptotic proteins Bax, Bcl-2, Cleaved caspase-3 and the LC3 puncta accumulation in the transfected groups compared with the control group cultured in normal medium (Fig. 4C,4D and 4E), suggesting inhibition of CLIC4 alone has no effect on cell death.

**Figure 5:**
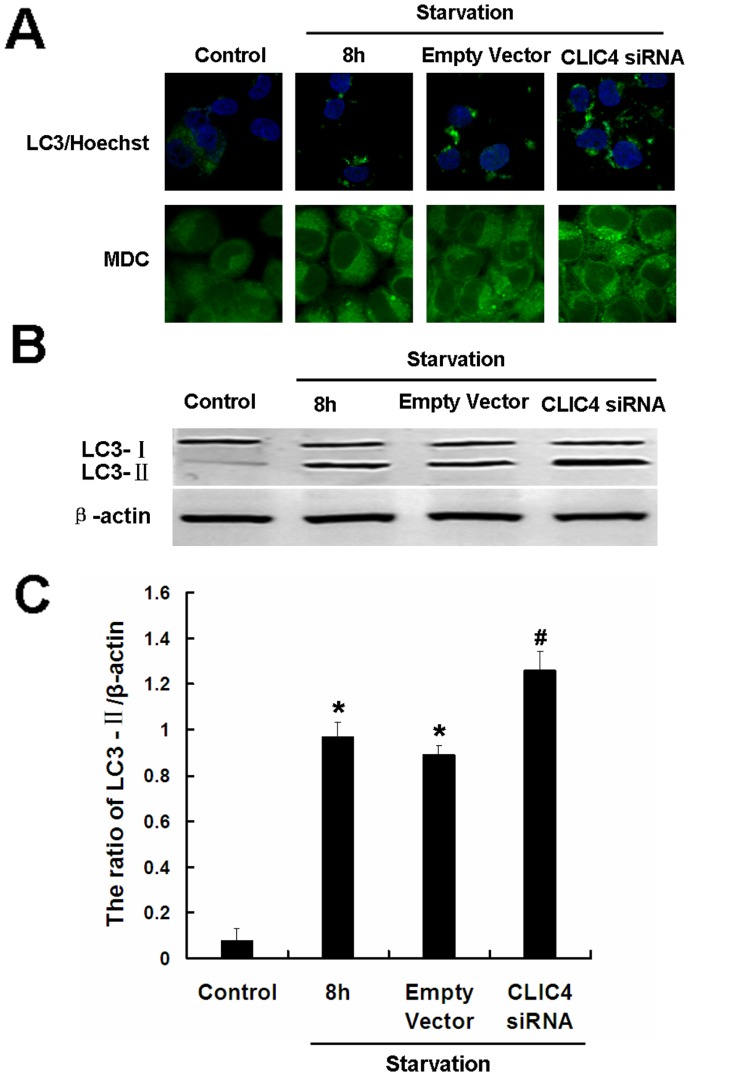
Inhibition of CLIC4 by siRNA enhanced autophagy under starvation conditions in U251 cells. U251 cells were transfected with a plasmid encoding empty vector or CLIC4 siRNA vector for 24 h. (A) LC3 immunostaining (Hoechst 33342 staining for the nucleus, 600×) and MDC staining (900×) were observed by confocal microscopy in U251 cells incubated with EBSS for 8 h. (B) Western blot analysis of LC3 expression in U251 cells incubated with EBSS for 8 h. (C) The ratio of LC3-II/β-actin. Data were presented as a mean ± SD of three independent experiments. ^*^ *P*<0.05 versus control group and ^#^ *P*<0.05 versus starvation group.

Next, we investigated the effect of CLIC4 siRNA on cell death including both autophagy and apoptosis under starvation conditions. LC3 and MDC staining showed increased puncta dots in the CLIC4 siRNA-transfected group incubated with EBSS for 8 h compared with the starvation 8 h group (Fig. 5A). Similarly, LC3-II protein expression was upregulated by CLIC4 siRNA transfection compared with the starvation group (Fig. 5B and 5C). These data indicate that partial inhibition of CLIC4 by siRNA enhanced autophagy in U251 cells under starvation conditions.

**Figure 6:**
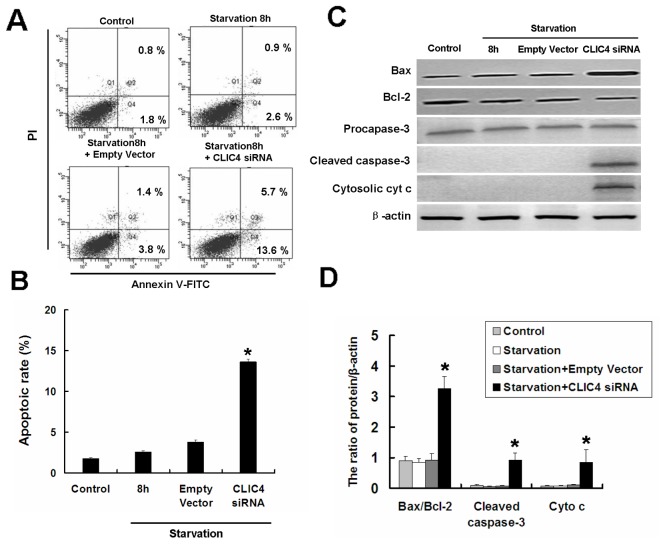
Inhibition of CLIC4 by siRNA triggered apoptosis under starvation conditions in U251 cells. U251 cells transfected with a plasmid encoding empty vector or CLIC4 siRNA vector for 24 h. (A) and (B) U251 cells incubated with EBSS for 8 h, then stained with PI and Annexin V-FITC to determine apoptotic rate. Data were presented as mean ± SD, n = 3. (C) Western blot analysis of Bax, Bcl-2, Cleaved caspase-3 and Cytosolic cyt c expression in U251 cells treated by EBSS for 8 h. (D) Densitometric analysis of normalized Bax/Bcl-2, Cleaved caspase-3 and cyt c levels. Standard error represents three independent experiments. *P<0.05 versus starvation 8 h group.

Since CLIC4 is associated with stress-induced apoptosis converging on the mitochondria, we next asked whether CLIC4 siRNA resulted in apoptosis activation under starvation conditions. The cell apoptotic rate was determined by PI/Annexin-V staining. No obvious apoptosis was evident in both the starvation and empty vector groups cultured with EBSS, whereas inhibition of CLIC4 by siRNA increased cell apoptotic rate significantly under starvation conditions compared with the control group (Fig. 6A and 6B). The apoptotic proteins Bax, Bcl-2, Caspase-3 and Cytochrome c (cyt c) were analyzed by immunoblotting. The ratio of Bax/Bcl-2, Cleaved caspase-3 and Cytosolic cyt c increased in starved CLIC4 siRNA-transfected U251 cells compared with the control starvation group (Fig. 6C and 6D). Altogether, these findings revealed the CLIC4 protein plays a prominent role in autophagy and apoptosis in U251 cells under starvation.

### Autophagy Enhanced by CLIC4 siRNA Transfection is Associated with Beclin 1 Activation Mediated by the 14-3-3 Epsilon

**Figure 7:**
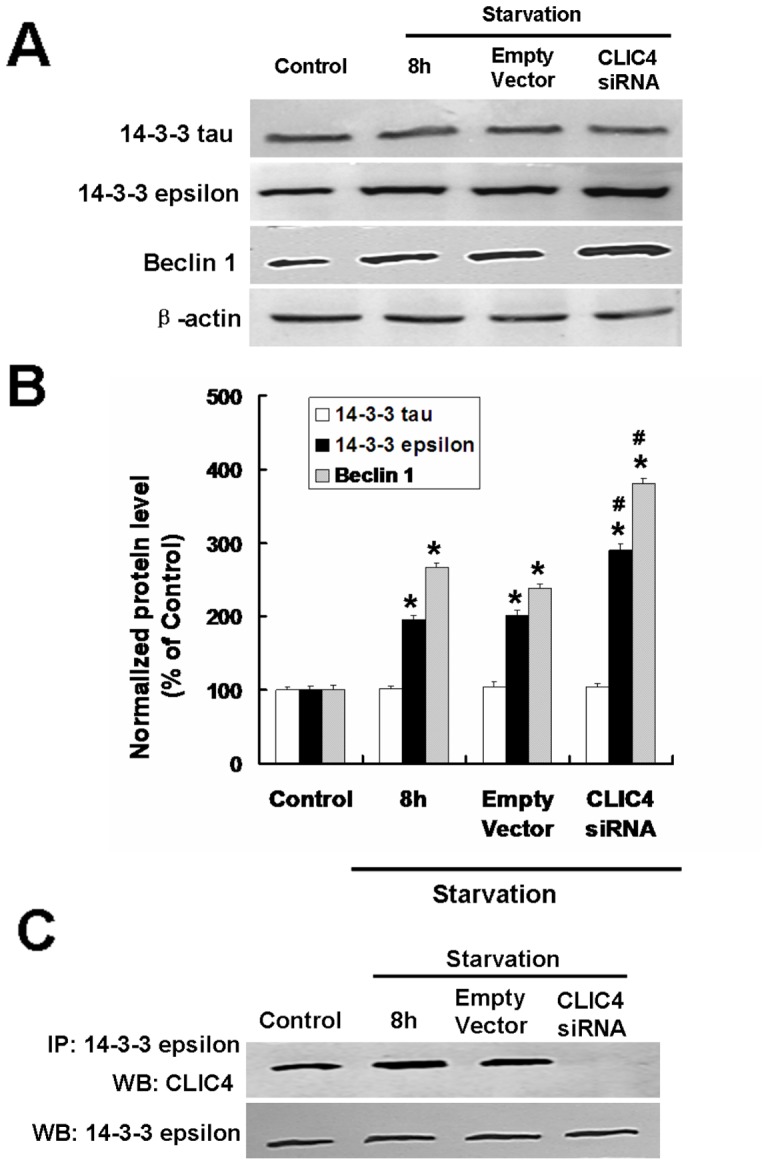
Enhanced autophagy by CLIC4 siRNA transfection is associated with interaction between Beclin 1 and 14-3-3 epsilon. U251 cells transfected with a plasmid encoding empty vector or CLIC4 siRNA vector for 24 h. (A) Western blot analysis of 14-3-3 tau, 14-3-3 epsilon and Beclin 1 expression in U251 cells incubated with EBSS for 8 h. (B) Densitometric analysis of normalized 14-3-3 tau, 14-3-3 epsilon and Beclin 1. Standard error represents three independent experiments. ^*^ *P*<0.05 versus control group and ^#^ *P*<0.05 versus starvation group. (C) Co-immunoprecipitation of 14-3-3 epsilon and CLIC4 in U251 cells under starvation conditions.

The 14-3-3 protein family contains seven mammalian isoforms (β, ε, γ, ζ, η, σ, τ), involved in many cellular processes, such as cell cycle control, signal transduction, vesicular transport, DNA repair and apoptosis [23]. Latest studies indicate that the 14-3-3 protein family is related to the regulation of Beclin 1, which is an essential part of the class III PI3-kinase complex that plays a central role in the induction of autophagy [24]. As the CLIC4 bands correlate directly to several of the 14-3-3 isoforms, we suspect that CLIC4 may be an indirect regulator of Beclin 1 activation in our model. To determine the molecular mechanisms among Beclin 1, the 14-3-3 isoforms and the CLIC4 protein, western blot and co-immunoprecipitation were applied. No significant difference in 14-3-3 tau protein expression, a required regulator in autophagy, was evident in human glioma U251 cells, even in the CLIC4 siRNA group under starvation conditions. This finding suggests that 14-3-3 tau may not be a regulator in our model and the role of 14-3-3 isoforms in starvation-induced autophagy may be distinct and cell specific (Fig. 7A). We next detected the level of the 14-3-3 epsilon, another 14-3-3 isoform indicated to bind with CLIC4 directly. Interestingly, the expression of the 14-3-3 epsilon increased in the EBSS group and was upregulated in the CLIC4 siRNA group under starvation conditions (Fig. 7A and 7B). The expression of Beclin 1 was also increased noticeably in the CLIC4 siRNA group under starvation conditions compared with the EBSS group, consistent with the expression of the 14-3-3 epsilon protein (Fig. 7A and 7B).

**Figure 8:**
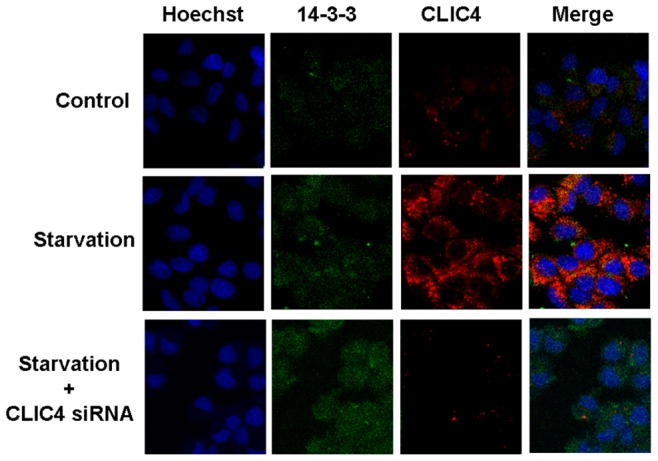
Inhibition of CLIC4 decreased co-localization of 14-3-3 epsilon and CLIC4 under starvation conditions. CLIC4 siRNA-transfected U251 cells for 24 h. Co-localization of 14-3-3 epsilon (green) and CLIC4 (red) were observed by confocal microscopy in U251 cells incubated with EBSS for 8 h (600×).

To further confirm the role of CLIC4 in starvation-induced autophagy and the relationship between 14-3-3 epsilon and CLIC4, co-immunoprecipitation analysis was applied. Co-precipitation of CLIC4 with 14-3-3 epsilon increased in the EBSS group (Fig 7C), indicating the role of CLIC4 in autophagy may relate to its interaction with the 14-3-3 epsilon. The interaction of CLIC4 protein with 14-3-3 epsilon was stopped by the CLIC4 siRNA transfection compared with the EBSS group. The interaction between CLIC4 and 14-3-3 epsilon is displayed by immunofluorescence staining. The co-localization of CLIC4 and 14-3-3 epsilon is indicated by the yellow signals in the composite images (Fig. 8). The co-localization increased significantly in the starvation group compared with the control group. The expression of CLIC4 (red fluorescence) was decreased by CLIC4 siRNA transfection and no co-localization with the 14-3-3 epsilon protein was evident (Fig. 8). These observations suggest the role of CLIC4 in starvation-induced autophagy may involve the interaction between the CLIC4 and 14-3-3 epsilon protein and activation of the autophagic gene Beclin 1, indirectly regulated by the 14-3-3 isoforms.

### CLIC4 siRNA Triggers ER Stress-induced Apoptosis Under Starvation Conditions

**Figure 9:**
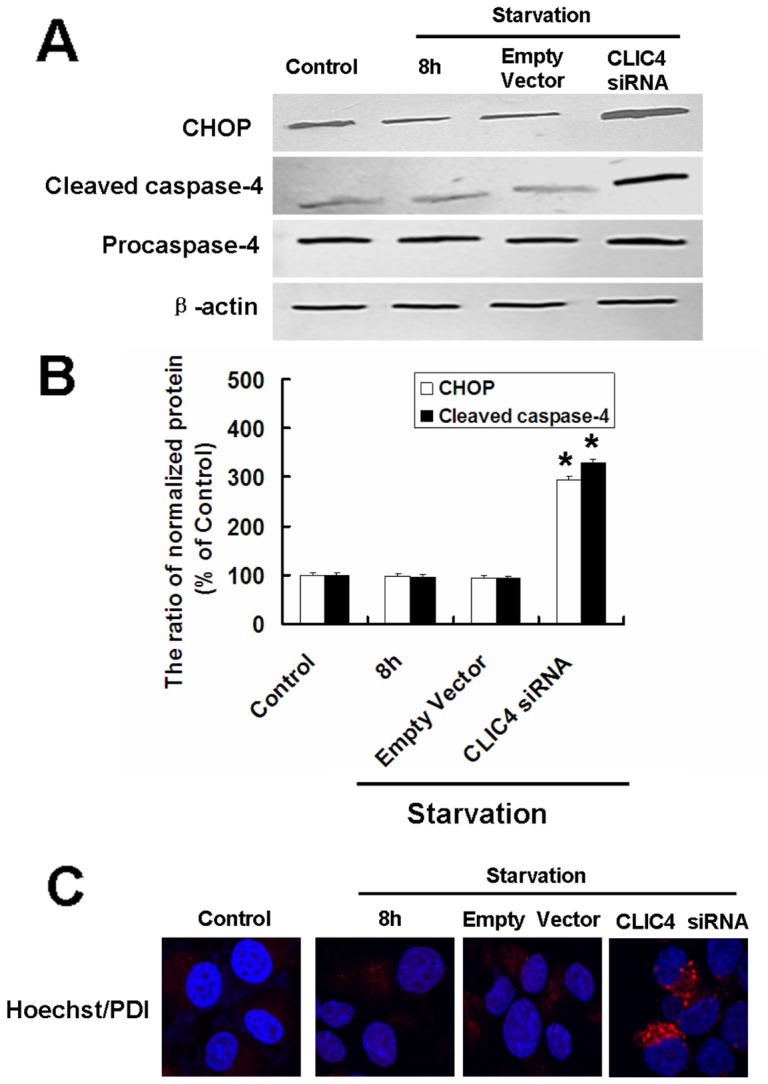
CLIC4 siRNA transfection triggered ER stress-induced apoptosis under starvation conditions. U251 cells transfected with a plasmid encoding empty vector or CLIC4 siRNA vector for 24 h. (A) Western blot analysis of CHOP, Procaspase-4, Cleaved caspase-4 expression in U251 cells incubated with EBSS for 8 h. (B) Densitometric analysis of normalized CHOP and Cleaved caspase-4. Standard error represents three independent experiments. ^*^ *P*<0.05 versus starvation 8 h group. (C) PDI immunostaining analysis of the ER stress activation (Hoechst 33342 staining for the nucleus, 900×) observed by confocal microscopy incubated with EBSS for 8 h.

Accumulating evidence indicates that ER stress is involved in apoptosis [25]. As CLIC4 may be localized at the ER membrane, we speculate that apoptosis induced by CLIC4 siRNA transfection may relate to ER stress activation. To address this, ER stress related apoptosis proteins CHOP and Caspase-4 were detected by western blotting. No obvious variation in the expression of CHOP and cleaved caspase-4 was evident in the starvation group compared with the control group. However, CLIC4 inhibition overactivated the ER stress in the starved cells and the levels of CHOP and Cleaved caspase-4 increased significantly compared with the starvation group (Fig. 9A and 9B). The ER luminal marker, protein disulfide isomerase (PDI) is considered an indicator of ER stress activation [26], [27]. Immunostaining with PDI showed a striking dilation in the ER of CLIC4 siRNA-transfected U251 cells under starvation conditions (Fig. 9C), indicating ER stress activation. Together, these results suggest ER stress along with CHOP and caspase-4 activation is associated with cell death following CLIC4 inhibition and nutrient starvation.

## Discussion

Malignant gliomas are the most common primary central nervous system (CNS) tumor of humans and are resistant to many kinds of conventional pro-apoptotic therapies, such as radiotherapy, chemotherapy and adjuvant therapies [28], [29]. Thus, developing novel strategies in malignant glioma therapy to induce non-apoptotic cell death, such as autophagy or cell death through mitotic catastrophe are required [30]. CLIC4 is highly conserved in different species with an amino acid sequence of nearly 95%, indicating its important role in cellular physiology. CLIC4 is widespread in the cytoplasm, especially in the intracellular membranes, such as the inner mitochondrial membrane and the ER [31]. CLIC4 has also been detected in various subcellular compartments including MV, cell–cell junctions, centrosomes, large dense core granules and nuclei among different cell types [16], [17], [32], [33], [34]. The function of CLIC4 is still unclear; accumulating evidence indicates CLIC4 plays an important role in many biological processes including signal transduction, cell differentiation and apoptosis [35]. However, the role of CLIC4 in autophagy has not been characterized.

In this study, the balanced salt solution EBSS was used to imitate the nutrient-free conditions to model classic autophagy. Our results show autophagy but not apoptosis was induced under starvation conditions in U251 cells. As a multifunctional and widespread protein, CLIC4 expression increased significantly in the EBSS incubated group, while starvation stress caused CLIC4 nuclear translocation. These findings suggest CLIC4 to be a potential responder or indirect regulator of autophagy. CLIC4 is susceptible to regulation; for example, CLIC4 was upregulated in the light-damaged retina and in endothelial cells treated with vascular endothelial growth factor [36], [37]. Thus, despite the name, CLIC4 may have a variety of discrete cellular functions in various physiological contexts. It has been shown that the nuclear translocation of CLIC4 has a global role in cellular physiology. Nuclear-targeted CLIC4 causes cyt c release from the mitochondria of primary keratinocytes. Endogenous CLIC4 translocates to the nucleus of Apaf null MEFs treated with DNA-damaging agents. CLIC4 nuclear translocation is a physiologic response through the interaction of a nuclear localization signal with nuclear transport proteins (Ran, importin and Nuclear transport factor 2) [16]. The function of CLIC4 in the nucleus has not been clarified at this time. The role of CLIC4 in autophagy may be associated with autophagic gene regulation by nuclear translocation or the interruption of protein-protein interactions. Furthermore, the presence of CLIC4 by nuclear translocation may participate in altering pH and chloride ion content that could be involved in pH relative events in the organelles, cytoplasm and nucleus [38]. It is worth mentioning that CLIC4 can also translocate to the plasma membrane upon receptor stimulation [39]. As acidic vesicular organelle formation is the main event in autophagy induction, we suspected CLIC4 upregulation and translocation might affect the acidification process through the indirect alteration of pH and chloride ion flux. However, these hypotheses require further investigation.

To confirm the role of CLIC4 in regulating starvation-induced cell death, CLIC4 siRNA was constructed and transiently transfected into the U251 cells. Interestingly, we observed autophagy under starvation conditions was enhanced along with an increased expression of both Beclin 1 and LC3-II protein levels. Study demonstrated one of the 14-3-3 isoforms, 14-3-3 tau is a required regulator in the expression of the autophagic gene Beclin 1, due to its role in the stabilization of the E2F1 transcription factor. Knockdown of 14-3-3 tau inhibited Beclin 1 protein expression and the LC3 puncta in nutrient limited conditions in both the human embryonic kidney HEK293 cells and carcinoma cell line HCT116 [24]. The 14-3-3 protein family plays a key regulatory role in signal transduction, checkpoint control, apoptotic, and nutrient-sensing pathways. The 14-3-3 proteins act by binding to partner proteins, often leading to altered subcellular localization of the partner. The 14-3-3 proteins are able to bind multiple phosphoserine-containing sequence motifs in different partners, such as Raf-1, Cdc25 and BAD, which promote the cytoplasmic localization of these proteins [40], [41], [42], [43]. In this study, we demonstrated that not the 14-3-3 tau but 14-3-3 epsilon, another 14-3-3 isoform which has been shown to interact with the CLIC4 directly, was upregulated under starvation conditions. Inhibition of CLIC4 by siRNA stopped the interaction of CLIC4 with 14-3-3 epsilon and resulted in an increase in Beclin 1. Our findings indicate that the role for CLIC4 in autophagy is related to the binding proteins crucial for CLIC4 in cell physiology, especially the 14-3-3 family isoforms.

Autophagy is a catabolic process providing an alternative energy source for cells under stressful conditions such as starvation. It was reported that apoptosis is induced by prolonging starvation time to over 48 h. With prolonged starvation and lysosomal inhibition, such cells acquired features of apoptosis including nuclear pyknosis and karyorrhexis [44]. These results suggested a possible shift from autophagy to apoptosis, yet were based on the utilization of pharmacological agents with limited specificity. LAMP2 (Lysosome-associated membrane protein 2) knockdown, which is essential for the fusion of autophagic vacuoles with lysosomes, sensitizes cells to starvation-induced cell death with autophagic and apoptotic characteristics by nutrient depletion of HeLa cells [45]. In *Drosophila*, developmental cell death in the salivary gland occurs in a stepwise fashion, with a caspase-independent, reversible autophagic morphology that drifts to a caspase-dependent, irreversible apoptosis [46], [47]. Thus, cells may ultimately succumb to biochemical processes such as caspase activation, which are typically associated with apoptosis. In this study, there was no significant apoptosis in the starvation group even at the 12 h time point; however, inhibition of CLIC4 by siRNA triggered apoptosis with an increase in Bax/Bcl-2 and caspase-3 activation under starvation conditions at the 8 h time point. The role for CLIC4 has been shown to assist with the pro-apoptotic gene *Bax* in the induction of apoptosis. Previous reports indicate an increase or reduction in CLIC4 impaired cell viability through an apoptotic pathway.

In addition to the mitochondrial apoptosis pathway, recent studies have demonstrated ER stress can be also involved in apoptosis [4], [25]. Various physiological and pathological conditions can interfere with protein folding in the ER, which can lead to ER stress. ER stress activates a complicated signal transduction pathway known as the unfolded protein response (UPR) [48]. The UPR can restore ER function following disruption. However, severe ER stress can cause cell death, usually by activating intrinsic apoptosis through upregulation of the ER stress-associated apoptosis proteins CHOP and Caspase-4 [49]. In the present study, we detected ER stress in CLIC4 siRNA U251 cells under starvation conditions, while CHOP and Caspase-4 activation indicated ER stress-induced apoptosis was another phase of cell death in our model. Despite being a multifunctional protein expressed on the ER membrane, the inhibition of CLIC4 under stress may cause a breakdown in ER homeostasis including the ion flux balance and stop unfolded protein degradation from the ER. The depletion of calcium and the accumulation of unfolded or misfolded protein will ultimately cause apoptosis, although further investigation is needed to confirm this hypothesis. Above all, our results indicate that inhibition of CLIC4 under starvation results in apoptosis of U251 cells. This is consistent with a study showing that suppression of CLIC family members, using an antisense approach, enhanced TNF-a-induced apoptosis in tumor cells [50].

In conclusion, the present study suggests that the CLIC4 protein plays an important role in autophagy and starvation-induced apoptosis in human glioma U251 cells. Nutrient-deprivation and hypoxia are common features of advanced solid tumors with autophagy, under these conditions, regulating cancer cell survival and drug resistance. Accordingly, manipulation of autophagy by inducing apoptosis has significant potential to improve efficacy of anticancer therapeutics [51]. In addition, the *CLIC4* gene is mapped to chromosome 1p36.11, a region that is frequently altered in cancers [50] and thus should be considered as potential molecular target for cancer therapy.

## Materials and Methods

### Cell Culture and Transfection

Human glioma U251 cells were purchased from the American Tissue Culture Collection (Rockville, MD). Cells were cultured under standard conditions in a humidified atmosphere in Dulbecco’s modified eagle media (Invitrogen, Carlsbad, CA) supplemented with 10% fetal bovine serum (FBS, Gibco, Grand Island, NY) at 37°C with 5% CO_2_. To simulate autophagy under amino acid starvation, glioma U251 cells were washed with PBS three times and then incubated in Earle’s balanced salt solution (EBSS, Sigma) at different time points (4 h, 8 h and 12 h) at 37°C with 5% CO_2_.

The plasmids were purified employing the HiSpeed Plasmid Maxi Kit (Qiagen Inc., Hilden, Germany). The Human CLIC4 siRNA (National Center for Biotechnology Information, accession numbers NM_013943 corresponded to the following cDNA sequence: 5-GCTGAAGGAGGAGGACAAAGA-3). The pSilencer 3.1 H1-neo plasmid was purchased from Ambion (Austin, TX). The U251 cells were cultured in six-well plates and transfected to obtain 80% confluence. Plasmids were transfected using Lipofectamine 2000 plus reagent (Invitrogen, Carlsbad, CA), according to the manufacturer’s recommendations. Western blotting was used to determine transfection efficiency. After 24 hrs, the transfected cells were ready for experimental use.

### Co-immunoprecipitation

The U251 cells were washed with cold phosphate-buffered saline (PBS) twice, then lysed using 1 ml radioimmunoprecipitation (RIPA) buffer (50 mM Tris-HCl [pH 6.8], 0.1% SDS, 150 mM NaCl, 1 mM EDTA, 0.1 mM Na_3_VO_4_, 1 mM sodium fluoride [NaF], 1% Triton X-100, 1% NP40, 1 mM dithiothreitol, and 1 mM PMSF, 1 µg/ml aprotinin, 1 µg/ml leupeptin, 1 µg/ml pepstatin A). After 30 min of lysis and centrifugation at 14,000×g for 15 min, the supernatants were collected. A 25 µl aliquot of protein A-Sepharose CL-4B (GE, Uppsala, Sweden) beads was added (50% in RIPA buffer) to immunoprecipitate the nonspecific antibody. Immune complexes were immobilized by adding 50 µl of protein A-Sepharose beads, washed three times with PBS, and 2 ×-sample buffer was added.

### Western Blotting

Cells were washed with PBS twice and harvested by scraping into 150 µl of RIPA buffer. Cell lysates were ultrasonicated for 15 s on ice and then lysed at 4°C for 45 min and centrifuged at 3,000 g for 20 min. Protein concentrations in the supernatants were determined by the BCA reagent (Thermo, MA). For Western blotting analysis, lysate proteins (60 µg) were separated by SDS-polyacrylamide gel electrophoresis (10–15%), transferred to nitrocellulose transfer membranes (Whatman, Maidstone, UK). The membranes were blocked with 5% nonfat dry milk in buffer solution (10 mM Tris-HCl [pH 7.6], 100 mM NaCl, and 0.1% Tween 20) for 2 h at room temperature and incubated with primary antibody (all purchased from Santa Cruz Biotechnology, CA) overnight at 4°C, followed by incubation with horseradish peroxidase–conjugated secondary antibody (Thermo, Waltham, MA) at 1∶1000 dilution for 2 h at room temperature. The immunoreactive bands were detected using the diaminobenzidine (Sigma, St. Louis, MO) coloration method. The protein was semiquantitatively analyzed with Tanon GIS gel imager System.

### Nuclear Protein Extraction

U251 cells were collected, washed with cold PBS and incubated in homogenization buffer (50 mM Tris-HCl pH 7.5, 0.8 M Sucrose, 150 mM KCl, 5 mM MgCl_2_, 6 mM β-mercaptoethanol, 0.5% NP-40 and protease inhibitor) for 10 min on ice [52], [53]. Supernatant was discarded and pellets were incubated in RIPA buffer by vortexing and sonication, followed by centrifugation at 16,000×g for 15 min at 4°C. Protein concentrations in the supernatants were determined by the BCA reagent (Thermo, MA). For Western blotting analysis, lysate proteins (60 µg) were separated by SDS-polyacrylamide gel electrophoresis (12%). The nuclear protein was normalized by nuclear marker Lamin as described before [16].

### Immunofluorescence Confocal Laser Microscopy

A fluorescent compound, MDC, has been proposed as a special tracer for autophagic vacuoles [22]. U251 cells were cultured on cover slips, treated as described above at different time points. After treatment cells were washed with PBS and stained with 50 µM MDC at 37°C for 1 h, fixed with 4% paraformaldehyde for 10 min and washed twice with PBS and immediately examined using the Olympus FV1000 confocal laser microscope.

The colocalizations of CLIC4 and 14-3-3 epsilon, and the expressions of protein disulfide isomerase (PDI) and LC3 were examined by indirect immunofluorescence. Cells were cultured on cover slips overnight, treated, and washed three times with PBS. After incubation, cells were fixed with 4% paraformaldehyde for 30 min, permeabilized with 0.1% Triton X-100 for 6 min, blocked with bovine serum albumin (BSA) and incubated with the primary antibodies CLIC4 and 14-3-3 epsilon, LC3 and PDI (1∶50 dilution) overnight at 4°C. Then the cells were incubated with FITC/Texas Red-conjugated secondary antibodies (1∶400 dilution) (all antibodies, Santa Cruz Biotechnology, CA) for 1 h, stained with Hoechst 33342 (2 µg/ml, Sigma) for 3 min, and washed three times with PBS and mounted onto glass slides. The cells were examined using the Olympus FV1000 confocal laser microscope.

### Flow Cytometry Analysis

Briefly, after exposure to the different conditions as described above, cells were incubated at 37°C for 15 min with Propidium iodide (PI, 1 µg/ml, Invitrogen) and Annexin V-FITC (1 µg/ml, Invitrogen) to determine cell apoptosis. The samples were analyzed by a FACScan flow cytometer (Becton Dickinson, Franklin Lakes, NJ).

### Statistical Analysis

Data are representative of at least three independent experiments performed in triplicate. Data were analyzed using a two-tailed Student’s t test. A *P* value <0.05 was considered statistically significant.

## Supporting Information

Figure S1 **Analysis of CLIC4 expression in U251 cells transfected with CLIC4 siRNA.** (A) Western blot analysis of CLIC4 expression in U251 cells transfected with encoding empty vector, CLIC4-siRNA-S and CLIC4 siRNA vectors for 24 h (B) Densitometric analysis of CLIC4 levels. Data were presented as a mean ± SD of three independent experiments. *P<0.05 versus control group.
(TIF)

Figure S2 **Analysis of CLIC1 and CLIC5 expression in U251 cells transfected with CLIC4 siRNA for 24** **h and 48** **h.**
(TIF)
