# Supplementary figures and images for: Inhibition of CLIC4 Enhances Autophagy and Triggers Mitochondrial and ER Stress-Induced Apoptosis in Human Glioma U251 Cells under Starvation
Source: PLoS One. 2012 Jun 25;7(6):e39378. doi: 10.1371/journal.pone.0039378 (PMC3382619; doi:10.1371/journal.pone.0039378)

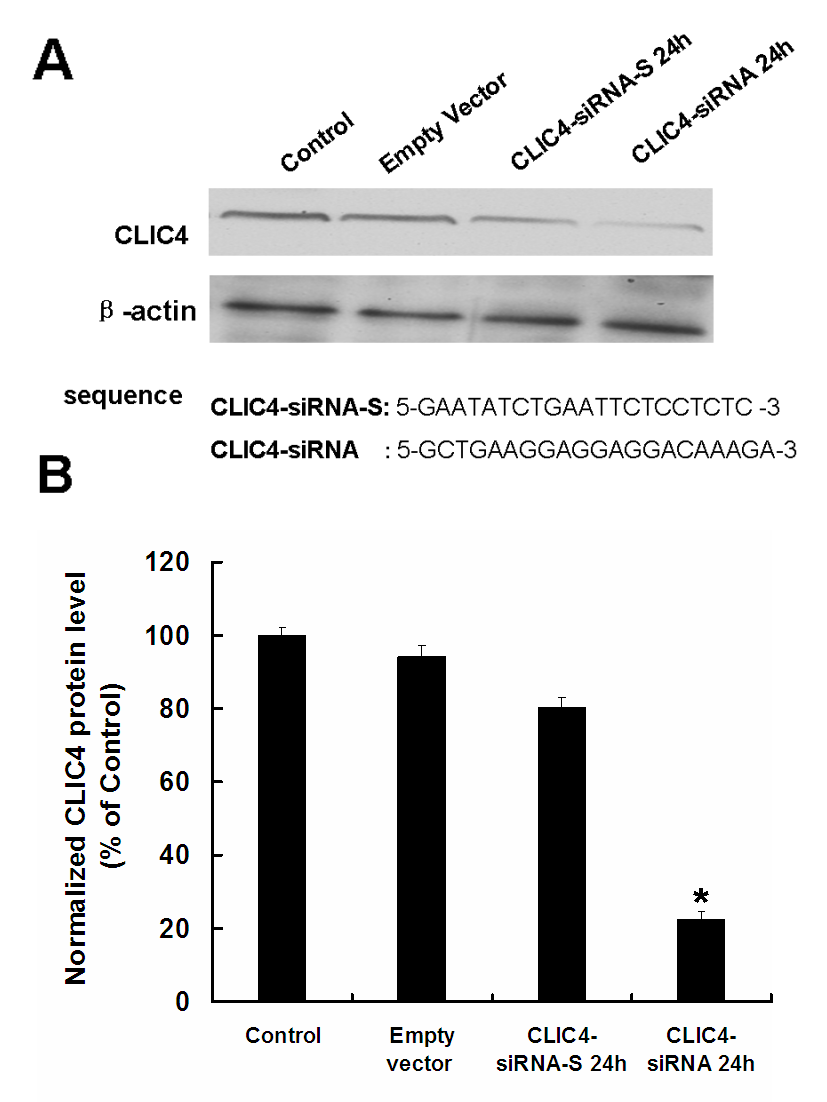

Supplement: Figure S1 — Analysis of CLIC4 expression in U251 cells transfected with CLIC4 siRNA. (A) Western blot analysis of CLIC4 expression in U251 cells transfected with encoding empty vector, CLIC4-siRNA-S and CLIC4 siRNA vectors for 24 h (B) Densitometric analysis of CLIC4 levels. Data were presented as a mean ± SD of three independent experiments. *P<0.05 versus control group. (TIF) [file pone.0039378.s001.tif]

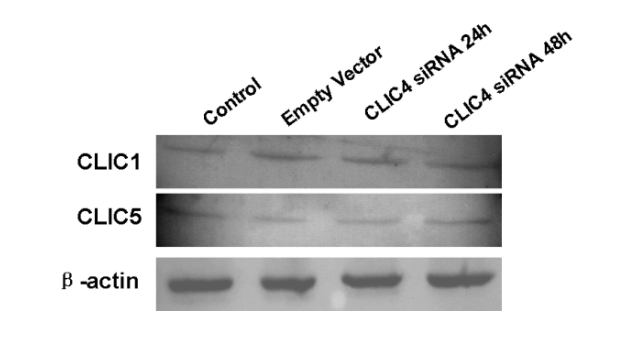

Supplement: Figure S2 — Analysis of CLIC1 and CLIC5 expression in U251 cells transfected with CLIC4 siRNA for 24 h and 48 h. (TIF) [file pone.0039378.s002.tif]
